# Remission from Alcohol Use Disorder among Males in the Lundby Cohort during 1947–1997

**DOI:** 10.1155/2018/4829389

**Published:** 2018-12-16

**Authors:** Cecilia Mattisson, Mats Bogren, Vibeke Horstmann, Leif Öjesjö, Louise Brådvik

**Affiliations:** ^1^Institute of Clinical Sciences Lund, Department of Psychiatry, Lund University, Baravägen 1, 221 85 Lund, Sweden; ^2^Department of Health Sciences, Faculty of Medicine, Lund University, 221 00 Lund, Sweden

## Abstract

**Background:**

Alcohol use disorders are a major health problem, often with a chronic course. Studies on remission from alcohol use disorders are sparse.

**Objective:**

The aim of this study was to analyse the rate of remission from AUD and the possible influence of other mental disorders and sociodemographic factors on the remission in the Lundby Cohort.

**Method:**

Remission from AUD was studied for 312 male subjects in the Lundby Cohort, which was followed for 50 years. Cox regression analyses were used to study the possible influence of sociodemographic variables and other mental disorders on AUD remission.

**Results:**

In all, 64/312 (21%) subjects achieved remission during the study period. The presence of a severe mental disorder, such as delirium tremens and organic disorders, was related to remission. Blue-collar workers had higher rates of remission than white-collar workers. There was indication that treatment improved the prognosis.

**Conclusions:**

The overall remission rate was low, but treatment may improve the prognosis. Severe mental disorders, such as delirium tremens and organic disorders as well as being blue-collar rather than white-collar worker, were related to remission.

## 1. Introduction

Alcohol use is a major contributor to disease, injury, and mortality [1]. However, alcohol use changes with age [2].

Some studies have reported remission rates of 40-60%, especially among high functioning alcoholics who were followed until age 50 and beyond [2–5]. Earlier findings from the Lundby Study reported a remission rate of 39%, in a subset of males with first incidence alcoholism who were followed for 40 years [6]. A 10-year follow-up study has reported a 30% remission rate in a community sample of outpatient health care recipients [7]. The National Epidemiological Survey on Alcohol and Related Conditions (NESARC study) reported a substantial remission rate from alcohol dependence of 29.6% [8] and an overall remission rate of 44% was reported for a sample of middle-class men followed for over 25 years [2].

Successful adjustment in alcohol use disorders could be found in atypical abusers, social drinkers, and abstainers [9]. Long-term studies on remission from alcohol use disorder (AUD) in general populations are rare and comparisons between studies are difficult due to variations in methodologies [10]. Considerable differences exist among definitions of recovery and remission in epidemiological studies; this contributes to the variability of reported outcomes of addiction treatment [11]. Addiction researchers often define the resolution of AUD in stages that range from abstinence to subclinical or socially accepted drinking habits. Clinical diagnostic criteria have sometimes distinguished between “abstinent recovery” and “nonabstinent recovery” regarding alcohol use [8].

The duration of time after drinking cessation required for study participation also varies among different studies [12]. Most studies have defined recovery in terms of remission rates among adults in the general population who did not meet substance use disorder criteria during the past year. A follow-up of subjects who achieved remission from alcohol dependence without treatment revealed that most untreated remissions with duration of at least 12 months were stable [13]. Other researchers suggest that 6-month remissions appear to be stable [14].

Furthermore, AUD is commonly termed a co-occurring disorder or part of dual diagnoses, and individuals with other mental disorders have high rates of AUD [15], even up to one-third in an Australian study [16]. On the other hand, persons with AUD often develop mental disorders, such as depression and anxiety [1]. Moreover, dual diagnoses have been associated with negative outcomes, such as relapses of mental disorders and continued alcohol abuse, with an increased risk of mood, anxiety, and personality disorders [17].

Sociodemographic factors have been associated with remission from alcohol dependence. A study investigating untreated remitters found that subjects receiving low social support were more likely to be in unstable natural recovery [18]. Also, McCutcheon et al. [19] found that environmental influences predominate in remission from AUD. Moreover, remissions were reported to be preceded by increased participation in substance abuse treatment [14]. General trend predictors for a poor prognosis in alcohol-related disorders can according to Öjesjö be arranged in the following order: severity of alcohol involvement most important, then age, work, and interpersonal relations [20].

In the present study, we aimed to investigate the rate of remission from AUD for males in the Lundby Study. We examined whether the occurrence of mental disorders was related to remission and we compared those subjects that had reached remission with those that did not. Contacts with healthcare are analysed for subjects in remission and nonremission individuals. We restricted the analyses to males since very few (35) of the subjects with alcohol use disorder were females in the Lundby Cohort.

## 2. Material and Methods

### 2.1. The Lundby Cohort

The Lundby Study is a prospective longitudinal study of an unselected population consisting of 3,563 individuals. It is a study of mental health in a geographically defined population [21], and it started in 1947 when Essen-Möller and coworkers conducted a prevalence study of mental disorders among the 2,550 inhabitants aged 0-92 years in the Lundby area in the south of Sweden [22]. Experienced psychiatrists conducted semistructured interviews and carefully described the subjects. The Lundby area consisted of two adjoining parishes at about 20-km distance from the old university town Lund. The population was thus geographically defined from the beginning of the study in 1947.

The first follow-up occurred in 1957; at that time 1,013 people, who had moved into or had been born into the Lundby area, were added to the original cohort, which brought the total number of subjects to 3,563 (1823 males/1740 females). No additional subjects have been added since 1957, but all individuals were assessed at later follow-ups regardless of where they lived. At the second follow-up in 1972 there were 2,827 survivors that were investigated by two psychiatrists. In the third follow-up in 1997, 1,797 survivors were investigated [21]. Information about those who had died between the field investigations was gathered throughout the time of follow-up. Medical records were gathered, and key informants were asked about the health of those who had died; certified causes of death and dates of death were obtained from the Swedish national cause of death registers (Epidemiological Centre, the national Board of Welfare). In 1947, the Lundby area was rural, and many farmers, farm labourers, and self-employed artisans lived there. Since the 1950s, about 50% of the survivors have moved, though many remained in neighbouring regions. The area has gradually changed into a suburban society from which inhabitants commute to work in neighbouring cities or villages.

The attrition rate has been low in the Lundby Study at follow-ups in 1947, 1957, and 1972, ranging between 1.2 and 1.8%. The dropout rate in 1997 among living subjects was higher (13%, 238/1,797), but it was still quite low.

### 2.2. Surveys

A semistructured interview resembling a clinical assessment was conducted at each time point. The interview was modernized in 1997 but kept its basic form throughout the study. One section of the interview was structured to generate information about episodes of mental disorders. The other part was unstructured, but often provided additional, valuable information (Hagnell et al., 1990). The majority of the interviews (1559) took place in the participants' homes or place of employment, but 128 telephone interviews out of the 1559 were performed in the 1997 field investigation, mainly due to distance and travel limitations.

Each interview began with questions about the subjects' physical and mental health since the previous investigation. Particular attention was paid to their contact with primary and psychiatric care, as well as hospital admissions. This section also explored alcohol problems and drug abuse. The CAGE-instrument was applied in the 1997 field investigation [23]. The interview assessed somatic illnesses and complaints, medications, smoking habits, sleeping problems, and appetite, as well as social life, important relationships, and general life satisfaction. Additional information was obtained through relatives, caregivers, and other key informants, such as general practitioners and local vicars. Official registers were also used: hospital case notes (psychiatric and nonpsychiatric); inpatient register and outpatient clinic [24]; county temperance board, which was operational until 1983; official death certificates from parish and central population registration; National Central Bureau of Statistics; and the cause of death register [25]. After information from these sources was gathered, diagnostic evaluations of mental disorders, including AUD, were conducted, and dates of emergence and remission were recorded. Information from the semistructured interviews, as well as from the county temperance boards and registers, were of considerable value for the diagnostic assessments. The final diagnostic assessments were performed within the research group after gathering of information from all available sources resulting in best estimate consensus diagnoses. Age at onset of AUD was assessed mainly from field investigations, participants, and/or key informants. Case files were important for the assessment of age at onset. Remission (stable recovery) was assessed from field investigations with additional data from other sources, such as medical records, registers, and key informants.

This information was also obtained for those who were dead at the different field investigations and data was obtained from all available sources.

### 2.3. Definitions of AUD and Remission

AUD includes alcohol abuse and alcohol dependence, as outlined in the Diagnostic and Statistical Manual of Mental Disorders, version IV (DSM-IV) [26]. An individual was assessed as having AUD if he or she met the DSM-IV criteria at any time between 1947 and 1997: (a) alcohol abuse is defined as a persistent pattern of recurrent alcohol use resulting in failure to fulfill major role obligations at work, school, or at home; i.e., the subject may use alcohol even in a physically hazardous situation as driving a car; there may be recurrent alcohol-related legal problems such as arrest for alcohol-related disorderly conduct; and the alcohol misuse is persistent despite social and relation problems caused by the effects of alcohol misuse. There were 127 males diagnosed with alcohol abuse.

(b) Alcohol dependence is defined by DSM-IV criteria with a maladaptive pattern of alcohol use, leading to clinically significant impairment or distress, such as intoxications and tolerance development and a withdrawal syndrome for alcohol may also be present. The definition of alcoholism in the Lundby Study is similar to the criteria in DSM-IV. The participants were asked about drinking patterns and impairment due to alcohol disorder at each field investigation. The Lundby interview comprises questions about how often and how much the participant drank. Further the amounts of beer (different kinds), wine, and liquor were asked for and if the participants have had problems with alcohol and if so of which kind. Tolerance, increasing use of alcohol, black-outs, and requirement for picker-upper were asked for. Moreover, in the Lundby Study AUD is usually identified by several sources of information as key informants, local temperance boards, registers, and medical records. In addition, an estimated duration of AUD of at least one year was required. The definition of remission was defined as having met none of the AUD criteria for at least one year.

There were 185 males diagnosed with alcohol dependence.

We have not used the categories abstinent remission and nonabstinent remission since we lack comprehensive information about drinking habits among the subjects during the whole 50-year follow-up. In a previous qualitative study the overall functioning was assessed by the Global Assessment of Functioning (GAF) scale and implied a change from continuous to transient symptoms and problems in the group that had achieved remission [10].

### 2.4. Socioeconomic Status

Socioeconomic status (SES) was defined from each individual's occupation and type of employment before the occurrence of incident AUD. In 1997, all subjects of working age at all follow-ups were rechecked according to the principles of Swedish SES classification (SEI code, [27]).

(1) Self-employed businessmen, artisans, and farmers.

(2) Middle-class employees or “white collar”.

(3) Working class or “blue collar”.

 Nonworking dependents were considered to be members of the social class of their caretakers. For students, the father's social class was used. If retired, the occupation participants had pursued for most of their working lives was used. Unemployed participants were categorized according to their most recent occupation. Housewives were classified according to their husband's SES.

### 2.5. Diagnoses of Mental Disorders

The Lundby Study started before the DSM was established and before structured diagnostic instruments were widely used. Therefore, the study facilitators used a simplified diagnostic system that is practical and adapted to fieldwork [21]. In this study, the main diagnostic categories applied were depressive disorders, anxiety disorders, psychotic disorders; alcohol-induced psychotic disorders, other psychotic disorders (mainly schizophrenia and bipolar disorders), organic disorders, and dementia.

AUD was assessed at the 4 different time points but also subsequently during follow-up. The diagnoses of alcohol-related psychotic disorders, primarily alcohol-induced psychotic disorders and delirium tremens, were obtained from inpatient register and hospital records. Organic syndrome included cognitive deficits, such as memory difficulties, slow reaction time, and concentration difficulties. Dementia included Alzheimer's disease, multi-infarct dementia, and other types of dementia. There was also a diagnostic category of mixed neuroses, in which no neurotic symptom was especially dominant, including neurotic states with symptoms such as fatigue, anxiety, depression, and obsessive-compulsive symptoms. In the last field investigation in 1997, DSM-IV diagnoses and ICD-10 diagnoses were assessed simultaneously with the Lundby diagnostic system.

### 2.6. The AUD Cohort

The Lundby Cohort comprises 1823 males and 1740 females. Only 35 women were diagnosed with AUD, and they were therefore excluded. The AUD cohort consists of 312 male subjects who were identified with AUD during follow-up 1947-1997 and with known age at onset of AUD. The duration of an on-going AUD that had started before the subject entered into the Lundby Cohort was determined for 103 cases out of 312 while 209 developed AUD during follow-up. An individual entered into the AUD cohort either when he entered the Lundby Cohort or at the day his AUD was assessed, whatever was the latest day. It is important to note that the 103 subjects entering the AUD cohort with an on-going period of AUD were not under risk of remission the first years until they entered the Lundby Cohort: an individual who recovered from AUD before entrance into the Lundby Cohort could by definition not be part of the present study. Thus, the follow-up period started at entry into the Lundby Cohort for the 103 who had AUD and at onset of AUD for the remaining 209. Of the 312 males with AUD 235 died during follow-up. There were 127 cases of alcohol abuse and 185 cases of alcohol dependence.

The sociodemographic characteristics of the subjects from the AUD cohort are presented in Table 1. Contacts with healthcare, including inpatient care, and temperance board are presented in Table 2.

### 2.7. Predictors of Remission in the AUD Cohort

Variables that were considered to be possible predictors of remission included age at onset, gender, SES, and diagnoses of mental disorders. Of the 312 subjects with AUD, 164 (52.6%) were diagnosed with a psychiatric disorder. The most common diagnoses were depressive disorders, observed in 60/312 (19.2%) subjects, and anxiety disorders, observed in 33/312 (10.6%) subjects. There were 12 subjects with some form of psychotic disorder with the most common diagnoses being schizophrenia and bipolar disorders. A total of 24 subjects were diagnosed with alcohol-induced psychotic disorders and delirium tremens.

### 2.8. Statistical Analysis

Descriptive statistics were used to present the sample. Chi–square tests were used to study differences in occurrence of treatment contacts and differences in occurrence of additional diagnoses of mental disorders between those who achieved remission and those who did not. Cox regression analyses were carried out with time from entry into the AUD cohort to remission as the outcome variable; the subjects were censored when death or end of study occurred before remission. Diagnoses of mental disorders were treated as time-dependent variables, coded as 0 before onset of the disorder and as 1 after the onset. First, we consider what we call simple Cox regression models where the influence of each mental disorder was assessed separately, controlling for age at entry into the AUD cohort, duration of AUD before entrance into the AUD cohort, and socioeconomic status. Thereafter multivariate regression analyses were performed starting with models including all mental disorders as possible risk factors, and where factors with nonsignificant contribution were omitted one by one in a stepwise manner. Also, all these models were adjusted for age at entry into the AUD cohort, duration of AUD before entrance into the cohort, and socioeconomic status (SES). Results were considered statistically significant when P < 0.05. The Statistical Package for the Social Sciences (SPSS), version 22, was used to analyse the data.

## 3. Results

### 3.1. Subjects Who Achieved Remission

Of the 312 subjects 64 subjects (21%) achieved remission during the study period. Thirty-four of 127 subjects (27%) with alcohol abuse remitted during follow-up and 30/185 (16%) with alcohol dependence reached remission. Subjects diagnosed with alcohol abuse achieved remission significantly more often than those subjects with alcohol dependence (Fisher's exact 2-sided test= 0.032).

The median age at remission was 48 years. For individuals who were observed to reach remission from AUD, the average duration of alcohol abuse was 6 years, while duration of alcohol dependence was 23.5 years. A total of 160 subjects died during the study period, and an additional 5 males were lost to follow-up for other reasons.

Table 2 lists subjects' contacts with health care services and temperance boards according to remission status. Of the 64 subjects who achieved remission 55 subjects had been admitted for inpatient care during the whole study period; subjects who achieved remission were significantly more likely to have been or to be admitted to inpatient care than subjects who did not reach remission (85.9% versus 64.5%; p<0.001). Subjects who reached remission had significantly more contacts with health care services of than subjects who did not recover (100% versus 87.9%; p<0.003). 17 out of 64 males in remission had been admitted to psychiatric care, whereas 52 out of 248 subjects not in remission had been or were to be admitted to psychiatric care (26.6% versus 20.9%). No difference was found between the different groups regarding their contact with a temperance board for corrective action.

Table 3 lists the frequencies of mental disorders present during the whole study period among all subjects according to remission status. There were no statistically significant differences in the presence of the mental disorders between subjects who achieved remission and those who did not achieve remission.

### 3.2. Predictors of Remission

Table 4 presents the results of regression analyses of factors that potentially influence remission. The simple regression models showed that remission was associated with a short duration of AUD before entry into the AUD cohort and lower SES. The remission rate was highest in working class (p-value= .038), which was statistically significant, somewhat lower but not significant for the self-employed (p-value= .544), compared to the white-collars (reference group). Organic disorder, delirium tremens, and substance-induced psychotic disorders were positively related to remission. Dementia, anxiety disorders, depressive disorders, and mixed neuroses did not affect remission significantly.

Multivariate models showed significant predictors of remission to be delirium tremens, substance-induced disorders (p-value=.016), blue-collar workers (p-value=.037), and organic disorders (p-value=.007).

## 4. Discussion

First the present study showed that the remission rate was relatively low (21%) despite a wide definition of remission, not only including abstainers. There was a significant difference in remission rates between abuse and dependence, and the time before remission was much longer in case of dependence (28 versus 6 years). We also found that severe psychiatric diagnoses, such as substance-induced psychotic disorders, organic disorder, and dementia, were predictors of remission. The analyses suggested that inpatient care and contact with the health care system could be positively associated with remission.

When comparing the remission rates in the Lundby Study with other studies that have reported higher remission rates it is important to bear in mind that these results can be based on drinking habits later in calendar time [3]. In general, there exist more available treatment facilities today. More longitudinal studies are needed to examine different drinking patterns and examine the effects on health throughout the life course [28].

Duration of remission required for study participation may vary between studies influencing the results and which makes comparisons difficult. Our study required one-year duration of remission which was rather long; also, this could contribute to the low remission rate in the present study. Abstinence from alcohol for 6 months may not indicate stable remission and relapses after 1 year are not uncommon [29]. High rates of relapse have been reported among young adults, indicating different risk of recurrence by age [3]. However, relapse was rare after abstinence had been maintained for 5 years, according to one study [30]. The same author concluded that, in contrast, return to controlled drinking without eventual relapse was unlikely. Other studies report that, among participants with a co-occurring severe mental disorder, two-thirds relapse at an average of 3 years after remission [14]. In that study subjects with alcohol dependence compared to alcohol abuse were less likely to attain 6-month remission which is similar to findings in the present study. Similar results were reported by Rapsey et al. 2018 [31] who reported slow transition from AUD to remission. Further, the authors emphasized that 50 % of dependent cases had not remitted after 9 years.

An explanation of the low remission rate in the present study in comparison to other studies may be that the Lundby Study had access to other sources of data, which provided more diagnostic information than was obtained from participants themselves. Some participants claimed they were not drinking when at the same time there were signs of actual drinking, for instance from the temperance board and thus the subjects were not considered in remission.

The Lundby area was in the beginning of the study in the forties and fifties rural, moonshining was more prevalent than nowadays, and many participants had alcohol disorders early in life. In rural settings such as in the Lundby area there could thus have been more tolerance for hazardous drinking. The Swedish authorities have tried to restrict harmful drinking. In 1919 a rationbook system was implemented on a national level where people over 20 years (except married women) were given an allowance of 1-4 litres of alcohol per month. This rationing system was abolished in 1955. Afterwards a dramatic increase in alcohol consumption is followed by a rise in various forms of alcohol-related harms; public drunkenness, rates of alcoholism, and alcohol psychoses doubled between 1954 and 1956 [32]. These changes in alcohol policy are likely to have influenced our remission rates as well. Many participants started drinking during the period with the rationbook system and are likely also to have increased their drinking after the system was abolished. Finally, less efficient treatment was available at the time, possibly indicating a better prognosis with the treatment of today.

Predictors of remission from AUD may be different from predictors of onset of alcoholism. Several factors have been suggested to be positively related to remission: finding a nonpharmacological substitute for alcohol, supervision, new relationships, and involvement in spiritual programs. Moreover, age at first use of alcohol had been shown to be a powerful predictor of lifetime alcohol abuse and dependence in the National Longitudinal Alcohol Epidemiologic Survey [33]. Turning points in a subject's life can trigger remission if they are positive and involve a heightened awareness and a cognitive-emotional shift in which the individual's regular pattern of seeing, interpreting, and approaching things was suddenly changed leading to motivation to achieve recovery and remission [34]. A previous study reported that abstinence decreased significantly with increasing educational level for both genders [30]. Our analyses showed that blue-collar status was positively related to remission and the relationship reached statistical significance when comparing to white-collar workers. Earlier research from the Lundby Study found that social stabilisation, unspecific treatment, family, peer pressure, and medical complications were important attributions for remission [10]. Further the role of social connection in remission from AUD has been documented in a wide variety of samples [35]. In the present study findings suggested that contact with health care could be a positive factor for achieving remission.

In the current study anxiety disorders, depressive disorders and other neurotic conditions did not affect remission rate significantly but more severe psychiatric diagnoses, such as substance-induced psychotic disorders; organic disorder and dementia were predictors of remission. The positive relation between delirium tremens and a favourable long-term outcome is in agreement with a previous study, in which delirium at first admission was related (Nordström & Berglund 1988). The result suggested in that study, though not statistically significant, that those having a depressive disorder were less likely to achieve remission. Comorbidity matters for many reasons [36]: persons with AUD and other mental disorders often have poorer treatment response and a worse course of illness suffering more impairment and greater social disability. Other studies have shown a relation between alcohol use disorders and depressive disorders [37] and anxiety disorders [38], but no significant associations were found in the present study, maybe due to sampling size and longer follow-up.

Subjects with a history of delirium tremens and organic syndromes were more prone to achieve remission in the present study. Subjects with psychotic disorders, dementia, and cognitive impairment may be so dysfunctional that continuing drinking is practically impossible. These findings agree with other research which states that problematic alcohol consumption may lead to health problems incompatible with further alcohol use [39]. It has been hypothesised that physiological processes of normal ageing with concomitant changes in reactions to alcohol may be of importance for remission from alcoholism in middle-aged and older alcoholics [40, 41]. There exist probably many reasons for changes in drinking pattern with age: changing life-cycle patterns, parenthood, changing social roles, and career goals.

The median age for remission in the present study was 48 years. The concept of ‘alcoholism as a self-limiting disease' [42] with a tendency to resolution after the age of 40 is in line with the present findings. Furthermore, for males by age 70, chronic alcohol dependence was rare due to both mortality and stable abstinence [30]. Subjects with very severe mental disorder could also be under care in nursing homes where alcohol use could be restricted. Such circumstances could affect remission rate. An intervention study suggests that inpatient treatment could be a window of opportunity for self-change and abstinence [43]. In the present study, most of the subjects who achieved remission had been admitted to inpatient care and psychiatric inpatient care. Significantly fewer subjects from the nonremitted group had been admitted to care, which suggests that inpatient care may be an important way to remission. In a long-term study on successful outcome in alcoholism, 60% of former patients stated that treatment had been of importance, in agreement with the present findings [9].

However, inpatient care may also indicate somatic or psychiatric complications, which may force subjects to stop drinking.

“Forced help” or compulsory treatment may be difficult to give to an unmotivated subject with AUD. In this study, no positive relation was detected between remission and contact with the local temperance board. However, one study showed that short-term treatment was successful for subjects suffering from alcohol-related problems of relative low severity and subjects appreciated more time with a therapist in this study [44]. Given that AUD is one of the most important risk factors for morbidity and mortality in several populations it remains a difficult challenge to promote remission.

### 4.1. Strengths and Limitations

The Lundby Study is not a clinically based study, which diminishes the probability of selection bias. Subjects in the AUD cohort comprises both treated and untreated persons course and provides a possibility of studying the natural course. The long follow-up period and the use of sources of information other than the individuals themselves such as registers, key informants, county temperance board, and medical records enrich knowledge of the participants. Another strength is the low attrition in the Lundby Study as well as the interviewer-assisted modes of data collection suggesting good quality of survey data [45]. A limitation is that the cohort is rather old. Also, changing diagnostic systems are problematic to handle during follow-up. The Lundby Study started before structured instruments were widely applied and thus a standardised validated interview was not used. DSM-IV diagnoses have only been applied in the period from 1972 to 1997, and other DSM diagnoses were added in retrospect. Further, when subjects in remission and not recovered are being compared as in the Tables 2 and 3 it is important to bear in mind that contacts with health care as well as presence of mental disorders could have occurred both before and after remission from AUD and it is not possible to conclude from these analyses that the factors could be predictors of remission. The same reservation does of course not hold for the psychiatric diagnoses in the Cox regression analyses where only mental disorders occurring during follow-up were considered.

## 5. Conclusion

The low remission rate of 21% in the present study emphasizes that AUD is a long-term disorder. This result can be explained by the fact that the treatment was less available in an earlier era represented in the study. The result could also have some resemblance with” natural course”. Estimates were also made from several sources, maybe giving more true rates than self-report. Remission was positively related to working class status and to some serious mental disorders, such as delirium tremens, substance-induced psychotic disorders, organic disorders and dementia. Inpatient care and a diagnosis of alcohol abuse were also positively related.

## Figures and Tables

**Table 1:** Demographic characteristics of males with known age at onset of AUD, n=312, at entry into the AUD cohort and males without AUD.

	Total N=312	Total without AUD N=1433
Age in years (Median, q1-q3)	31(20-44)	30(12-46)
SES classification, n (%)		
Blue-collar workers	240 (77.0)	632 (62.9)
White-collar workers	42 (13.5)	105 (10.53)
Self-employed	29 (9.3)	267 (26.6)
Marital state, n (%)		
Unmarried	68 (22)	776 (54.2)
Married/cohabitate	175 (56.1)	607 (42.4)
Divorced	50 (17.3)	9 (0.6)
Widow/widower	19(6.1)	41 (2.9)

Notes: q1-q3, Quartile 1-Quartile 3; SES, socioeconomic status. For males without AUD there was no SES information for 429 males, because children were not categorised into the socioeconomic classification.

**Table 2:** Previous contacts with healthcare and temperance board for subjects with AUD who reached remission n=64 and subjects who did not remit n=248. Any kind of contact with health care refers to all kinds of treatment facilities including stay in nursing homes.

	Not remitted N=248	Remitted N=64
Contact with outpatient clinics, n (%)	88 (35.5)	25 (39.1)
In patient-care*∗*	160 (64.5)	55 (85.9)
In-patient psychiatric care	52 (20.9)	17 (26.6)
Any contact with healthcare*∗*	218 (87.9)	64 (100)
Contact with Temperance Board	116 (46.7)	30 (39.1)

Note: observe that participants can have had contact with various health care facilities.

Note: contact with health care facilities could have occurred prior to or after recovery.

*∗*Significant differences between subjects who recovered and subjects who did not recover were detected by Chi-square tests.

**Table 3:** Frequencies of mental disorders in subjects in remission and those who did not remit from AUD for 312 males with known age at onset of AUD. Percentages are given in parentheses. P-values refer to testing the equalities in proportion of mental disorder for those who achieved remission and those who did not.

Mental disorders, n (%)	Not remitted N=248	Remitted N=64	P-values
Anxiety disorders	9 (14.1)	24 (9.7)	0.309
Depressive disorders	10 (15.6)	50 (20.2)	0.412
Mixed neurosis	5 (2.0)	2 (3.1)	0.593
Organic syndrome	5 (7.8)	9 (3.6)	0.149
Dementia	6 (9.4)	18 (7.3)	0.571
Psychotic disorders	2 (7.7)	10 (4.0)	0.737
DT and alc-induced*∗*	6 (9.4)	18 (7.3)	0.571

DT: delirium tremens; alc-induced: alcohol-induced psychotic disorder. Chi-square test showed no significant differences between subjects in presence of mental disorder. Note: mental disorders could have occurred prior to or after remission.

**Table 4:** Simple and multivariate Cox regression analyses of predictors of remission from AUD among participants with known age at onset of AUD.

	All N=312		
	HR	CI	P
**Simple models**			
Age at entry	1.02	1.00-1.03	0.009
Duration of AUD	0.87	0.80-0.94	0.001*∗*
SES			0.062
White-collar	1.0		
Blue-collar	2.94	1.06-8.15	0.038*∗*
Self-employed	1.54	0.38-6.20	0.544

Anxiety disorders	1.46	1.08-3.06	0.323
Depressive disorders	1.45	0.69-3.11	0.294
Mixed neurosis	1.65	0.40-6.80	0.489
Psychotic disorders	0.72	0.17-2.94	0.643
DT and substance induced	3.30	1.34-8.11	0.009*∗*
Organic syndrome	4.85	1.64-14.3	0.004*∗*
Dementia	3.80	0.86-16.8	0.078
Alcohol dependence	1.81	0.78-4.14	0.163

**Multivariate models**			
Age at entry	1.01	0.99-1.03	0.085
Duration of AUD	0.87	0.80-0.94	<0.001
SES			0.035*∗*
White-collar	1.0		
Blue-collar	2.96	1.07-8.21	0.037*∗*
Self-employed	1.24	0.30-5.10	0.77
DT and subst ind	3.03	1.23-7.46	0.016*∗*
Organic disorder	4.47	1.50-13.30	0.007*∗*
Dementia	4.21	0.95-18.7	0.059

The outcome variable is time to remission from entry into the AUD cohort. Simple models included only one of the risk factors mental disorders and the variables: duration of AUD before entrance into the cohort, age at entry into the AUD cohort, and SES. White collar was considered a reference category. Psychotic disorders refer to schizophrenia and bipolar disorders. HR, hazard rate; CI, 95% confidence interval; AUD, alcohol use disorder; SES, socioeconomic status; DT, delirium tremens; substance-ind, substance (mostly alcohol)-induced psychotic disorders.

## Data Availability

The data used to support the findings of this study are available from the corresponding author upon request.
